# Development and validation of the creatinine clearance predictor machine learning models in critically ill adults

**DOI:** 10.1186/s13054-023-04553-z

**Published:** 2023-07-06

**Authors:** Chao-Yuan Huang, Fabian Güiza, Pieter Wouters, Liese Mebis, Giorgia Carra, Jan Gunst, Philippe Meersseman, Michael Casaer, Greet Van den Berghe, Greet De Vlieger, Geert Meyfroidt

**Affiliations:** 1 Laboratory of Intensive Care Medicine, Academic Department of Cellular and Molecular Medicine, KU Leuven, Leuven, Belgium; 2 Department of Intensive Care Medicine, University Hospitals Leuven, Leuven, Belgium; 3 Department of General Internal Medicine, Medical Intensive Care Unit, University Hospitals Leuven, Leuven, Belgium

**Keywords:** Creatinine clearance, Intensive care unit, Prediction model, External validation, Machine learning

## Abstract

**Background:**

In critically ill patients, measured creatinine clearance (CrCl) is the most reliable method to evaluate glomerular filtration rate in routine clinical practice and may vary subsequently on a day-to-day basis. We developed and externally validated models to predict CrCl one day ahead and compared them with a reference reflecting current clinical practice.

**Methods:**

A gradient boosting method (GBM) machine-learning algorithm was used to develop the models on data from 2825 patients from the EPaNIC multicenter randomized controlled trial database. We externally validated the models on 9576 patients from the University Hospitals Leuven, included in the M@tric database. Three models were developed: a “Core” model based on demographic, admission diagnosis, and daily laboratory results; a “Core + BGA” model adding blood gas analysis results; and a “Core + BGA + Monitoring” model also including high-resolution monitoring data. Model performance was evaluated against the actual CrCl by mean absolute error (MAE) and root-mean-square error (RMSE).

**Results:**

All three developed models showed smaller prediction errors than the reference. Assuming the same CrCl of the day of prediction showed 20.6 (95% CI 20.3–20.9) ml/min MAE and 40.1 (95% CI 37.9–42.3) ml/min RMSE in the external validation cohort, while the developed model having the smallest RMSE (the Core + BGA + Monitoring model) had 18.1 (95% CI 17.9–18.3) ml/min MAE and 28.9 (95% CI 28–29.7) ml/min RMSE.

**Conclusions:**

Prediction models based on routinely collected clinical data in the ICU were able to accurately predict next-day CrCl. These models could be useful for hydrophilic drug dosage adjustment or stratification of patients at risk.

*Trial registration*. Not applicable.

**Supplementary Information:**

The online version contains supplementary material available at 10.1186/s13054-023-04553-z.

## Background

Critical illness often affects kidney function. Epidemiologic studies have shown that 40–60% of intensive care unit (ICU) patients have episodes of acute kidney injury (AKI) [1, 2], whereas in 20–65% of the patients, days with an augmented renal clearance (ARC) occur [3]. Accurate assessment of kidney function is crucial for risk stratification and drug dosage adjustment, especially for renally cleared drugs such as vancomycin and β-lactam anti-microbials. Most often, kidney function is evaluated through serum creatinine, and renal clearance is estimated based upon the modification of diet in renal disease study (MDRD) [4], or Chronic Kidney Disease Epidemiology Collaboration (CKD-EPI) [5] formulas. However, these commonly used estimation formulas were derived from non-critically ill patients and thus have their limitations in properly estimating kidney function in the ICU setting [6–10], especially in long-stay patients [11]. Creatinine clearance (CrCl) measured from a 24-h urine collection reflects the kidney function in both reduced and augmented renal clearance in routine clinical practice [3, 11]. As kidney function may change rapidly in critically ill patients [12, 13], even a calculated CrCl based on the urinary CrCl of the past 24 h may lag behind true kidney function. Potentially, an accurate prediction of kidney function in the next 24 h could allow for more suitable therapeutic interventions.

Existing machine-learning predictions for kidney function have focused on predicting the onset of AKI [14–22], because of its high incidence [23] and strong associations with higher mortality, longer length of stay, and heavier financial burden [24, 25]. Other models predict ARC, which is commonly defined as the presence of CrCl greater than 130 ml/min/1.73 m^2^ and has significant consequences concerning the pharmacokinetics of hydrophilic drugs [26–30]. Studies have demonstrated that ARC patients need higher antibiotic doses [31], have more treatment failure [32], and have a doubled risk of subtherapeutic vancomycin serum concentrations [33]. AKI and ARC prediction models were based on categorized definitions. As the glomerular filtration rate (GFR) is in fact continuous, being able to predict the entire kidney function spectrum corresponds better with clinical and physiological reality.

Despite the importance and need for continuous kidney function prediction, to the best of our knowledge, no prediction models for daily prediction of CrCl in critically ill patients exist. Hence, this study aims to develop and validate prediction models that apply machine learning algorithms to routinely collected patient data to predict CrCl one day ahead.

## Methods

### Prediction tasks and CrCl definition

This study aims to predict the CrCl of the next patient day. CrCl was calculated by daily 24-h urine output (UO), urinary creatinine (UCr), and serum creatinine (SCr) without correction for an average body surface area: CrCl (ml/min) = UCr(mg/dL) × 24-h UO(ml/day) /SCr(mg/dL)/1440 (min/day). In an additional analysis, the same methodology was applied to develop models to predict the average CrCl over the next two days ahead (Additional file 1: Methods).

### Study databases with inclusion and exclusion criteria

The large multicenter EPaNIC randomized controlled trial (RCT) database [34], where two parenteral nutrition strategies were compared in 4640 critically ill adults between August 2007 and November 2010, was used for model development. This study was conducted on the basis of prior informed consent by all patients or their legal representatives, and the consent forms included the permission to use the data for additional research (S50404). For this secondary study, patients were eligible for inclusion if they had no kidney replacement therapy (KRT) before ICU. Patient-days were excluded if there were 1) no CrCl measurements on the next day, 2) no CrCl measurements on the day of prediction, 3) KRT on the day of prediction, 4) KRT in the previous week, and/or 5) all patient days beyond 90 days in the ICU were excluded. The CrCl was measured on a routine basis in all patients with a bladder catheter during the trial.

External validation was performed on a dataset of 20,930 patients of the University Hospitals Leuven included in the large multicenter M@tric database between 2013 and 2018 [35]. The M@tric database contains high-quality data from all adult patients admitted to the ICUs of the three largest university hospitals in Belgium. Ethical approval for the M@tric database collection was received from the Ethics Committee (EC) of University Hospitals Ghent. Approval for the use of these patient data in the present study was obtained from the EC of University Hospitals Leuven (S61364). The study was conducted in compliance with the principles of the Declaration of Helsinki and its later revisions. The same exclusion criteria as described above for the EPaNIC development dataset were applied. The CrCl was routinely measured in all patients with a bladder catheter in the surgical ICU, but not routinely in the patients from the medical ICU.

### Feature engineering

Only data up to the day of predicting CrCl were used as input to the models. The considered data included: 1) admission data: demographics, diagnosis, and comorbidities, 2) time-series data such as minute-by-minute monitoring data and daily or hourly laboratory results, 3) medication-related data, 4) time-related data: day of the week, and day from ICU admission. Data were retrieved from both the EPaNIC study database (Filemaker Pro®; FileMaker Inc, FileMaker International) and the patient data management system (PDMS) database (Microsoft SQL Server®; Microsoft®, Redmond, Washington, USA).

The minimum, maximum, mean, standard deviation, linear regression slope, fast Fourier transform (FFT), cepstrum analysis, autoregressive analyses, and first-order derivative were applied to derive characteristics from the timestamped data. All the features with more than 10% missing values were excluded. For the remaining features, missing values were imputed with the mean and the mode from the development cohort for continuous data and categorical data, respectively. Finally, continuous data were standardized to zero mean and unit variance, and categorical data without order relation were converted into a form with binary data for each category.

### Machine-learning algorithm, feature selection methods, and clinical prediction models

The prediction models were trained with the gradient-boosting regressor method [36], with features selected from the PDMS system by a backward elimination method [37], and thorough discussions with two experienced ICU physicians (GDV and GM). Hyperparameters were fine-tuned with Optuna hyperparameter optimization software [38].

For each prediction task, three models with progressively more features were developed which are meant to be utilized sequentially, based on the data availability at the bedside.

- A “**Core model**” using only admission data and daily routine laboratory results.
- A “**Core + BGA model**” that adds to the above, blood gas analysis data.
- A “**Core + BGA + Monitoring model**” that adds to the above, monitoring data (heart rate, mean arterial blood pressure, and respiratory rate).

### Internal and external validation

Models were developed and internally validated on the EPaNIC database with tenfold cross-validation. At the external validation stage, models trained on the entire EPaNIC database were applied to the previously unseen external validation cohort to assess generalizability. To examine the model usefulness, model performance was further compared against a reference reflecting the current clinical practice: using as prediction for one-day ahead the same CrCl value of the day of prediction. This reference CrCl was henceforth referred to as **Prediction day’s CrCl**. To assess daily fluctuations in CrCl, we calculated the difference between the CrCl of each pair of two consecutive days. It was labeled as stable if the absolute difference was less than 20 ml/min and unstable if more than 20 ml/min, as this is a meaningful difference for drug dosing and because the CrCl variability in healthy volunteers has been reported with mean differences of 21.7 ml/min/1.73m^2^ [39].

### Evaluation metrics for predictive performance

Mean absolute error (MAE) and root-mean-square error (RMSE) were computed for all available patient-days, stable days, and unstable days for each model in both cohorts. Both MAE and RMSE measured the errors between the model predictions and the target CrCl values, with RMSE more sensitive to large errors. Predictive performance was also evaluated visually with scatter plots and plots of daily MAE and RMSE for all available patient-days, stable days, and unstable days during the first week of ICU stay. As multiple patient-days were available in many patients, no overall p-value can be calculated as this may be biased by repeated measures, but we compared the MAE on a day-by-day basis with the Diebold–Mariano test [40]. Count-based feature importance of the developed models was visualized with bar plots.

### Descriptive analyses and software used

Python 3.7.4 (Python Software Foundation, http://www.python.org), SciPy version 1.3.1 (SciPy.org), and Scikit-learn library 0.24.2 (scikit-learn.org) were used for all analyses. The study population was described using descriptive statistics, with continuous data presented as medians and interquartile ranges (IQR) and categorical data expressed as counts and percentages (%). Mann–Whitney U test and Fisher's exact test were used to evaluate the statistical significance of differences for continuous and categorical data, respectively. Significance levels were set at the 5% level.

## Results

### Study cohorts

#### Development cohort

For the model development, data were retrieved from 2825 patients, equivalent to 18,494 patient-days (Additional file 1: Fig. S1). The descriptive statistics are shown in Table 1. Median (interquartile range, IQR) age was 67.6 (56.2–75.6) years, median (IQR) Acute Physiology and Chronic Health Evaluation II (APACHE II) score was 22 (16–32), the majority was with cardiac surgery (n = 1655, 58.6%), and median (IQR) average CrCl over the entire ICU stay was 93.5 (58.2–131.8) ml/min. The median ICU length of stay (IQR) was 5 (3–11) days, and 162 (5.7%) patients died before ICU discharge. There were 6371 (34.5%) unstable days.

**Table 1:** Patient characteristics and clinical outcomes

	Development cohort (*n* = 2825)	Validation cohort (*n* = 9576)	*p*-value
Age, years, median (IQR)	67.6 (56.2–75.6)	65.5 (54.6–75)	< 0.01
Gender male, number (%)	1747 (61.8)	5816 (60.7)	0.3
Mean creatinine clearance over the entire ICU stay, ml/min, median (IQR)	93.5 (58.2–131.8)	93.1 (56.2–133.6)	0.7
Emergency admission, number (%)	1272 (45)	3231 (33.7)	< 0.01
APACHE II score, median (IQR)	22 (16–32)	17 (13–21)	< 0.01
Reason for admission			
Cardiac surgery, number (%)	1655 (58.6)	3229 (33.7)	< 0.01
Medical disease, number (%)	114 (4)	2316 (24.2)	< 0.01
Neurology, number (%)	119 (4.2)	363 (3.8)	0.3
Trauma and other surgery, number (%)	662 (23.4)	2859 (29.9)	< 0.01
Transplantation, number (%)	275 (9.7)	809 (8.4)	0.04
ICU mortality, number (%)	162 (5.7)	200 (2.1)	< 0.01
Length of stay in ICU, days, median (IQR)	5 (3–11)	4 (2–9)	< 0.01

*ICU* intensive care unit; *APACHE II score* Acute Physiology and Chronic Health Evaluation II score

#### External validation cohort

For the external validation of the developed models, data from 53,943 patient-days from 9576 patients were used, corresponding to 45.8% of the University Hospitals Leuven patients included in the M@tric database. Compared to the development cohort, the age was younger (median (IQR) 65.6 (54.6–75) years, p < 0.01), emergency admission was less frequently (n = 3231, 33.7%, p < 0.01), APACHE II score was lower (median (IQR) 17 (13–21), p < 0.01), cardiac surgery was still the major admission diagnosis but occurred less often (n = 3229, 33.7%, p < 0.01), and average CrCl over the entire ICU stay was similar (median (IQR) 93.1 (56.2–133.6) ml/min, p = 0.7) (Table 1). The ICU length of stay was shorter (median (IQR) 4 (2–9) days, p < 0.01), and the ICU mortality was lower (n = 200, 2.1%, p < 0.01). There were 16,514 (30.6%) unstable days.

### Features selected for CrCl prediction

**Fig. 1:**
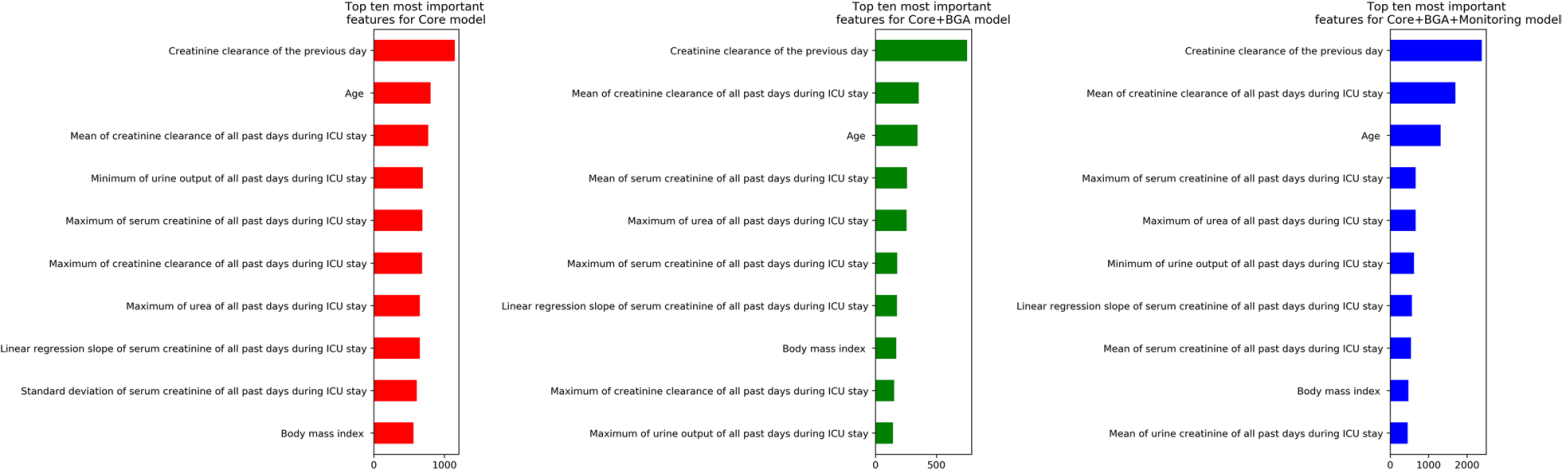
Top ten most important features of different models. The red, green, and blue bar plots are the results for the Core, Core + BGA, and Core + BGA + Monitoring models, respectively

Among the ten most predictive variables of the three models, seven were related to CrCl, one to urea level, and the remaining two were the baseline characteristics age and body mass index (BMI) (Fig. 1). For the three models, the top ten most important features were features already available in the Core model. In other words, neither BGA nor monitoring data related features were among the top ten most important features of any model. The full set of features was presented in Additional file 1: Results.

### Externally validated model performance

**Fig. 2:**
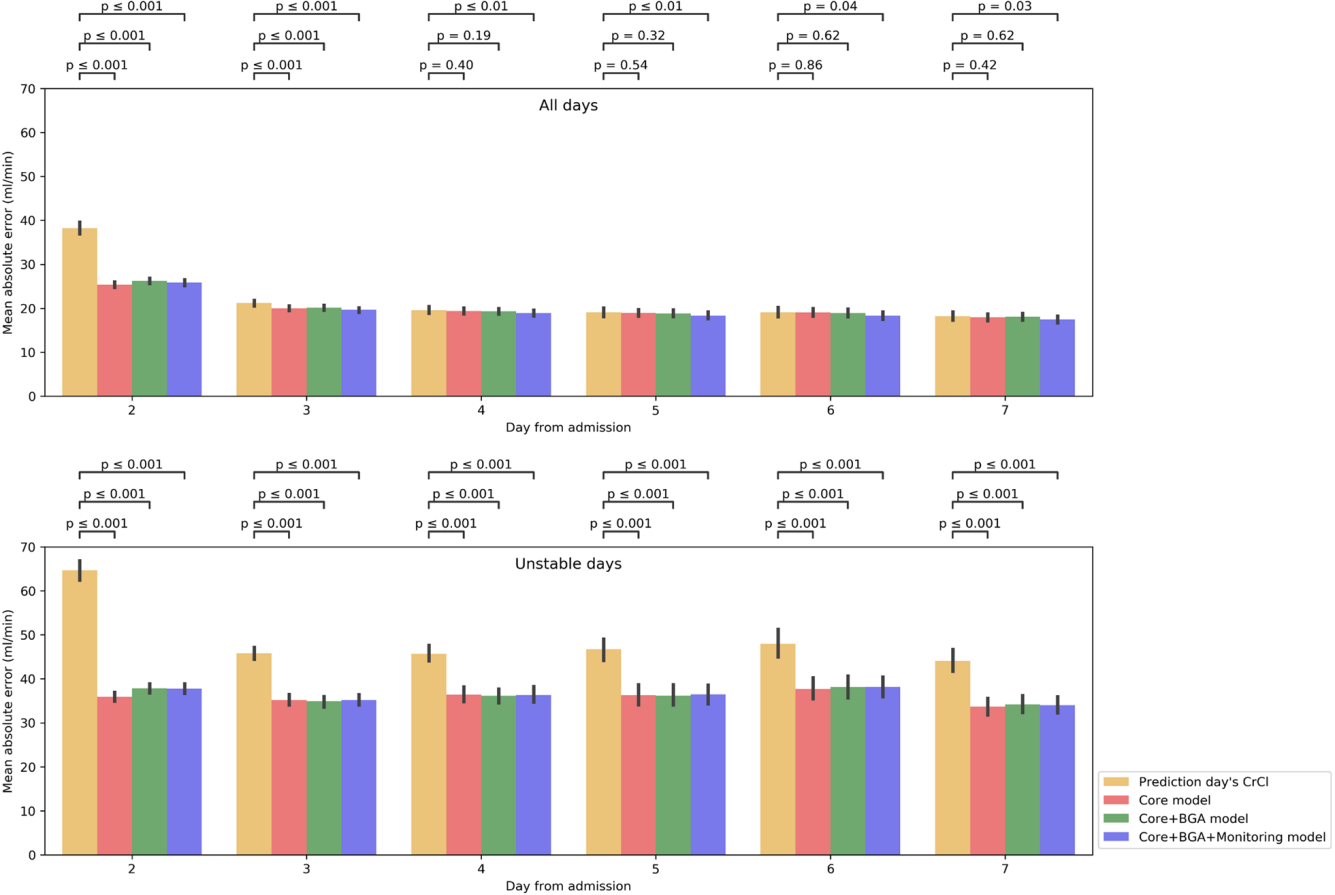
Temporal mean absolute error of different models on all days and unstable days within the first week of ICU admission in the validation cohort. The red, green, blue, and orange bars represent, respectively, the Core, Core + BGA, Core + BGA + Monitoring models, and the reference that assumes CrCl will remain the same compared to the day of prediction. Error bars represent 95% confidence intervals

**Fig. 3:**
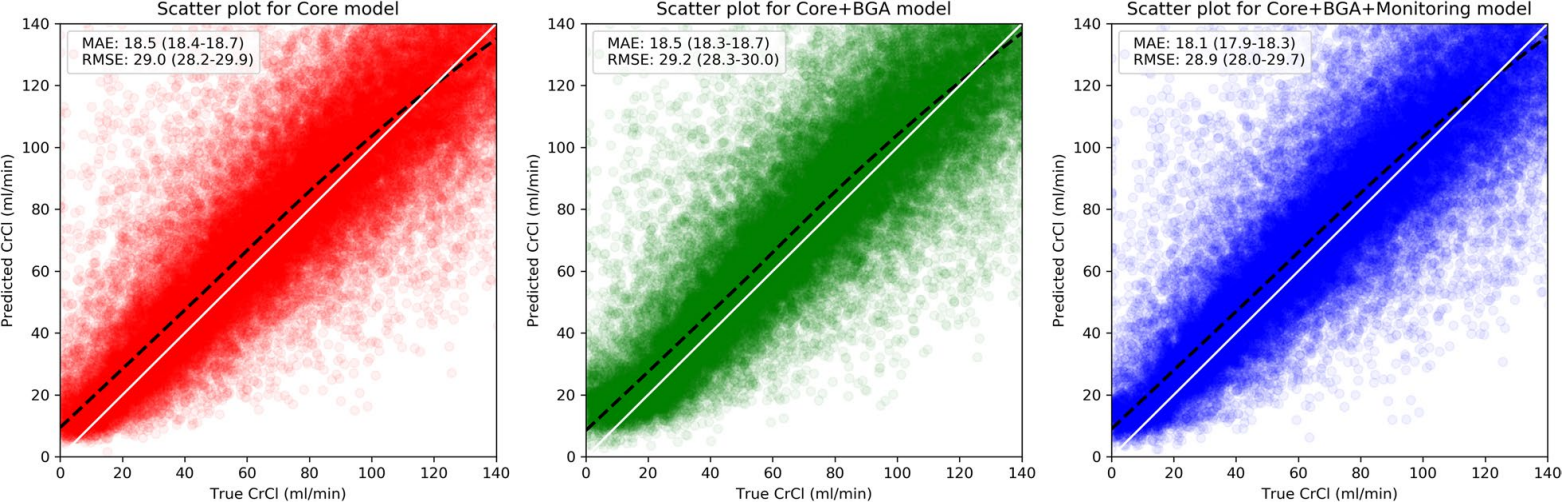
Relationships between predicted and actual CrCls for different models in the validation cohort. The red, green, and blue scatter plots show the results for the Core, Core + BGA, and Core + BGA + Monitoring models, respectively. The black dashed and white solid lines represent the lowess-based regression lines for the developed models and the diagonal axis. MAE, mean absolute error; RMSE, root-mean-square error; CrCl, creatinine clearance

The developed models performed well in both the internal validation (Additional file 1: Figs. S2 and S3, Table S1) and the external validation (Figs. 2 and 3, Table 2). Specifically, for all patient-days, the model having the smallest RMSE in the validation cohort was the Core + BGA + Monitoring model, which exhibited 18.1 (95% CI 17.9–18.3) ml/min MAE and 28.9 (95% CI 28–29.7) ml/min RMSE, while the prediction day’s CrCl led to a 20.6 (95% CI 20.3–20.9) ml/min MAE and 40.1 (95% CI 37.9–42.3) ml/min RMSE.

**Table 2:** Summary of mean absolute error and root-mean-square error for the developed models and the reference on all days, stable days, and unstable days in the validation cohort

	All days		Stable days		Unstable days	
	Mean absolute error (ml/min) (95% CI)	Root-mean-square error (ml/min) (95% CI)	Mean absolute error (ml/min) (95% CI)	Root-mean-square error (ml/min) (95% CI)	Mean absolute error (ml/min) (95% CI)	Root-mean-square error (ml/min) (95% CI)
Prediction day’s CrCl	20.6 (20.3–20.9)	40.1 (37.9–42.3)	7.6 (7.5–7.6)	9.3 (9.3–9.4)	50.1 (49.3–50.9)	71.1 (67–75.1)
Core model	18.5 (18.4–18.7)	29 (28.2–29.9)	11.4 (11.3–11.5)	15.1 (14.9–15.3)	34.8 (34.3–35.2)	47.3 (45.6–49)
Core+BGA model	18.5 (18.3–18.7)	29.2 (28.3–30)	11 (10.9–11.1)	14.6 (14.4–14.8)	35.4 (34.9–35.9)	47.9 (46.2–49.5)
Core+BGA+Monitoring model	18.1 (17.9–18.3)	28.9 (28–29.7)	10.5 (10.4–10.6)	13.9 (13.7–14.1)	35.5 (35–36)	47.9 (46.2–49.5)

*CrCl* creatinine clearance

During the stable days, the model having the smallest RMSE was the Core + BGA + Monitoring model, demonstrating 10.5 (95% CI 10.4–10.6) ml/min MAE and 13.9 (95% CI 13.7–14.1) ml/min RMSE, while the prediction day’s CrCl showed 7.6 (95% CI 7.5–7.6) ml/min MAE and 9.3 (95% CI 9.3–9.4) ml/min RMSE. The model had a larger RMSE of 4 ml/min on average than the prediction day’s CrCl.

However, on the days when kidney function was unstable, the model having the smallest RMSE was the Core model exhibiting 34.8 (95% CI 34.3–35.2) ml/min MAE and 47.3 (95% CI 45.6–49) ml/min RMSE, whereas the prediction day’s CrCl showed 50.1 (95% CI 49.3–50.9) ml/min MAE and 71.1 (95% CI 67–75.1) ml/min RMSE. The model had a smaller RMSE of 23 ml/min on average than the prediction day’s CrCl.

These differences in prediction performance for unstable days remained when analyzing the daily predictions during the first week of ICU stay only as evidenced in Fig. 2, where there was a significant difference in MAE between the Core + BGA + Monitoring model and the reference. When plotting predicted against actual CrCls (Fig. 3), a good agreement was observed, with most predictions located at or near the diagonal axis. The results of the two days ahead average CrCl predictions are discussed in Additional file 1: Results.

## Discussion

In this study, we presented three models to predict daily CrCl in critically ill adults, based on information derived from routinely collected clinical data. The predictive performance remained similar when adding high-resolution data. The developed models were externally validated on previously unseen patients with good performance. Finally, the models demonstrated smaller prediction errors than using the CrCl of the day of prediction (reflecting the current clinical practice. This is mainly explained by the better performance of the prediction model as compared to the reference of the previous day on the days with a bigger change in the CrCl (i.e., ‘unstable days’), whereas the model performed similarly as the reference of the previous days on the days that the CrCl remained stable (‘stable days’). To the best of our knowledge, this study presents the first machine-learning algorithm for daily CrCl prediction in the ICU.

There are many reasons why there is a need for such daily prediction of CrCl during the entire ICU stay. First, measured urinary CrCl is currently considered the most suitable method to estimate the GFR in clinical practice [41], as many studies have shown the limited ability of estimation methods in the ICU setting [6–9]. Second, several hydrophilic antibiotics are mainly eliminated by the kidneys, so dosage adjustment is necessary to prevent drug toxicity in reduced renal clearance patients [42] and treatment failure in ARC patients [32]. Third, a minimum of eight-hour time window of urine collection, but preferably 24 h, is necessary to ensure a reliable urinary CrCl measurement [43]. Consequently, the kidney function might have already changed by the time urine collection is complete. This delayed kidney function information could endanger patients by giving the physicians a false impression of kidney function when prescribing drugs, as we observed here that the strategy using the measured CrCl of the past 24 h led to large estimation errors (RMSE of 40.1 ml/min). Armed with these prediction models, clinicians may optimize drug dosing, and as such lower the risk of adverse effects, and improve overall patient outcomes. For instance, doses of antibiotics such as beta-lactams and vancomycin can be adjusted based on the predicted CrCl on the next day, which may result in achieving therapeutic targets and avoiding drug accumulation with associated drug toxicity.

Having a reference to compare against helps to understand whether the models could have clinical usefulness. Compared to the current clinical practice of assuming the same CrCl as the day of prediction, our developed models reduced the RMSE from 40.1 to 28.9 ml/min. Importantly, in the subgroup of patient-days with stable kidney function, the developed models demonstrated a clinically insignificant larger RMSE, around 4 ml/min on average, than the reference. This difference, however, is too small to be of clinical relevance and unlikely to cause treatment failure or drug toxicity due to altered renal clearance. Noticeably, in the subgroup of patient-days with unstable kidney function (comprising 30–40% of all patient-days), the developed models had clinically relevant smaller RMSEs, around 23 ml/min on average. This subgroup analysis of days with high CrCl instability clearly exhibited our models’ capability of better capturing the dynamics of kidney function. Nevertheless, despite the large reduction in prediction errors during unstable days, whether or not the models help improve drug dosing and patient outcomes still needs to be investigated prospectively.

Our study has many strengths. First, the use of a general ICU population instead of a specific subset of patients made it more generally applicable, and the daily prediction truthfully reflected the fluctuating kidney function on each patient day, allowing for risk stratification and drug dose adjustment. Second, the reporting of this study was performed following the Transparent Reporting of a Multivariate Prediction Model for Individual Prognosis or Diagnosis (TRIPOD) guidelines [44]. Third, both internal and external validation were performed, and the developed models were compared against a reference to fairly report the model performance and robustness without overoptimism. Fourth, not only static data but also timestamped data applied with advanced feature engineering techniques were progressively included with increasing data resolution. Finally, the use of a very large validation dataset of approximately 54,000 patient-days from over 9500 mixed critically ill patients attested to the robustness of the findings.

There are several limitations in our study. First, the development cohort was based on a RCT database in Belgium dating back to 2010, which might limit its generalizability in other settings. However, model performance remained unchanged when externally validated on a very large database with patient data collected up to 2018. Second, the use of high-resolution data might be difficult to implement in hospitals with limited resources, and some settings might even struggle to have the necessary data for the lower-resolution Core model. Third, there might be a selection bias resulting from the exclusion from the analyses of patient-days with KRT on the day of prediction and in previous week, or of patient-days when less than 2 consecutive CrCls were available, or patient-days after the first 90 days in ICU. These exclusion criteria were necessary to ensure reliable CrCl prediction models could be developed. Fourth, this study was based on retrospective data, and the developed models still need prospective validation in independent cohorts. Fifth, the model performance was not compared against novel biomarkers such as cystatin C and proenkephalin that may be less biased, and urea clearance that may add valuable information especially in those with low CrCl. However, measured CrCl is a fast and cheap test, which are important characteristics as the measurements were taken on a daily basis. Sixth, the measurement of creatinine changed from the Jaffe method in the development cohort to the enzymatic method in the validation cohort, and it was found that the Jaffe method yielded higher creatinine values [45]. However, the Jaffe and enzymatic creatinine methods were shown with adequate overall agreement (r = 0.9994 and 0.9998 in serum and urine, respectively), and thus, the influence of changed creatinine measurements was expected low. Finally, the developed models were not implemented as bedside tools, integrated into clinical practice, and transferred to other centers yet, but it was beyond the scope of this work and remains a challenging topic for future studies.

## Conclusions

We have shown that CrCl can be accurately predicted one day in advance during ICU stay. We have also demonstrated the robustness of the developed models on previously unseen patients in external validation. The developed models’ usefulness has been shown in comparison with a reference reflecting current clinical practice, mainly on the patient-days with high kidney function instability. Despite the promising performance, these findings should be prospectively validated in independent patient populations, before these prediction models can be further used for risk stratification or incorporated into a pharmacokinetic model to support a more optimized dose regimen.

## Supplementary Information

**Additional file 1:** Supplementary methods and results.

## Data Availability

The datasets generated and/or analyzed during the current study are not publicly available due to no prior agreement with the ethical committee but are available from the corresponding author on reasonable request.

## Abbreviations

CrCl: Creatinine clearance
GBM: Gradient boosting method
MAE: Mean absolute error
RMSE: Root-mean-square error
SCr: Serum creatinine
UO: Urine output
UCr: Urine creatinine
ICU: Intensive care unit
AKI: Acute kidney injury
ARC: Augmented renal clearance
MDRD study: Modification of Diet in Renal Disease study
CKD-EPI: Chronic Kidney Disease Epidemiology Collaboration
GFR: Glomerular filtration rate
RCT: Randomized controlled trial
KRT: Kidney replacement therapy
EC: Ethics Committee
PDMS: Patient data management system
FFT: Fast Fourier transform
IQR: Interquartile ranges
BMI: Body mass index
TRIPOD: Transparent Reporting of a Multivariate Prediction Model for Individual Prognosis or Diagnosis
FWO: The Research Foundation – Flanders
APACHE II score: Acute Physiology and Chronic Health Evaluation II score
CI: Confidence interval
