# Supplementary material for: Development and validation of the creatinine clearance predictor machine learning models in critically ill adults
Source: Crit Care. 2023 Jul 6;27:272. doi: 10.1186/s13054-023-04553-z (PMC10327364; doi:10.1186/s13054-023-04553-z)
Supplement: Supplementary file 1 — Additional file 1: Supplementary methods and results. [file 13054_2023_4553_MOESM1_ESM.pdf]

Supplementary Methods

Unstable days definition for prediction of average CrCl of two days ahead

For prediction of average CrCl of two days ahead, to assess daily fluctuations in CrCl, we calculated the difference between the CrCl of two consecutive days of three consecutive days. It was labeled as stable if either of the two absolute differences was less than 20 ml/min and unstable if both were more than 20 ml/min.

Machine learning algorithms

Several algorithms have been explored, including Gradient boosting machine, Random Forest, Logistic regression with L1 norm, K neighbors regressor, Gaussian processes regressor, Extra trees regressor, Huber regressor, Support vector machine regressor, Bayesian ridge, Kernel ridge regressor, Long short-term memory, Stacking of the abovementioned algorithms. In the end, we chose the gradient boosting method for the higher interpretability and decent predictive performance.

Supplementary Results

For the average CrCl of two days ahead prediction task, the majority of the predictions was located near to the diagonal axis, denoting a good agreement between predicted and actual CrCls (**Supplementary Fig. 4**). When analyzing the daily predictions during the first week of ICU stay only as shown in **Supplementary Fig. 5**, the models were with lower prediction errors for all days and unstable days. The worse MAE on stable days was not of clinical relevance as the difference was small, around 4 ml/min on average.

Complete feature list:

One-day-ahead prediction:

For the one-day-ahead Core model, a total of 26 features were used. 4 were derived from serum creatinine, 3 were derived from creatinine clearance, 3 were derived from medication/intervention (aminoglycosides medication on the day of prediction, number of days with respiratory support in all past days during ICU stay, and number of days with vasopressors/inotropes medication in all past days during ICU stay), 2 were derived from bilirubin, 2 were derived from urine output, 2 were derived from urine creatinine, 1 was derived from urea, 1 was derived from hematocrit, 1 was derived from c-reactive protein, 1 was derived from SOFA score, 1 was day of the week, 1 was gender, 1 was age, 1 was day from ICU admission, 1 was APACHE II score on the first day of ICU, and 1 was body mass index.

For the one-day-ahead Core+BGA model, a total of 65 features were used. 7 were derived from BGA pH value, 7 were derived from BGA bicarbonate, 6 were derived from BGA partial pressure of oxygen, 6 were derived from BGA lactate, 4 were derived from serum creatinine, 3 were derived from creatinine clearance, 3 were derived from medication/intervention (aminoglycosides medication on the day of prediction, number of days with respiratory support in all past days during ICU stay, and number of days with vasopressors/inotropes medication in all past days during ICU stay), 3 were derived from BGA sodium, 3 were derived from BGA potassium, 3 were derived from BGA partial pressure of carbon dioxide, 2 were derived from BGA glucose, 2 were derived from bilirubin, 2 were derived from urine output, 2 were derived from urine creatinine, 2 were derived from BGA hemoglobin, 1 was derived from urea, 1 was derived from hematocrit, 1 was derived from c-reactive protein, 1 was derived from SOFA score, 1 was day of the week, 1 was gender, 1 was age, 1 was day from ICU admission, 1 was APACHE II score on the first day of ICU, and 1 was body mass index.

For the one-day-ahead Core+BGA+Monitoring model, a total of 74 features were used. 7 were derived from BGA pH value, 7 were derived from BGA bicarbonate, 6 were derived from BGA partial pressure of oxygen, 6 were derived from BGA lactate, 5 were derived from heart rate, 4 were derived from serum creatinine, 3 were derived from creatinine clearance, 3 were derived from medication/intervention (aminoglycosides medication on the day of prediction, number of days with respiratory support in all past days during ICU stay, and number of days with vasopressors/inotropes medication in all past days during ICU stay), 3 were derived from BGA sodium, 3 were derived from BGA potassium, 3 were derived from BGA partial pressure of carbon dioxide, 2 were derived from BGA glucose, 2 were derived from bilirubin, 2 were derived from urine output, 2 were derived from urine creatinine, 2 were derived from respiratory rate, 2 were derived from mean arterial blood pressure, 2 were derived from BGA hemoglobin, 1 was derived from urea, 1 was derived from hematocrit, 1 was derived from c-reactive protein, 1 was derived from SOFA score, 1 was day of the week, 1 was gender, 1 was age, 1 was day from ICU admission, 1 was APACHE II score on the first day of ICU, and 1 was body mass index.

Average-of-two-days-ahead prediction:

For the prediction of average CrCl of two days ahead, a total of 67 features were employed in the Core model. 8 were derived from urea, 7 were derived from serum creatinine, 6 were derived from chloride, 6 were derived from platelet, 6 were derived from SOFA score, 4 were derived from urine output, 4 were derived from urine creatinine, 4 were derived from hematocrit, 4 were derived from creatinine clearance, 4 were derived from c-reactive protein, 3 were derived from white blood cell count, 2 were derived from medication/intervention, 2 were derived from bilirubin, 1 was age, 1 was weight, 1 was APACHE II score on the first day of ICU, 1 was transplant diagnostic group on ICU admission, 1 was gender, 1 was day from ICU admission, and 1 was day of the week.

For the prediction of average CrCl of two days ahead, a total of 130 features were employed in the Core+BGA model. 8 were derived from BGA partial pressure of oxygen, 8 were derived from urea, 8 were derived from BGA pH value, 7 were derived from serum creatinine, 7 were derived from BGA bicarbonate, 7 were derived from BGA sodium, 6 were derived from BGA hemoglobin, 6 were derived from chloride, 6 were derived from BGA potassium, 6 were derived from BGA lactate, 6 were derived from platelet, 6 were derived from SOFA score, 6 were derived from BGA glucose, 5 were derived from BGA partial pressure of carbon dioxide, 4 were derived from urine output, 4 were derived from BGA calculated oxygen saturation, 4 were derived from urine creatinine, 4 were derived from hematocrit, 4 were

derived from creatinine clearance, 4 were derived from c-reactive protein, 3 were derived from white blood cell count, 2 were derived from medication/intervention, 2 were derived from bilirubin, 1 was age, 1 was weight, 1 was APACHE II score on the first day of ICU, 1 was transplant diagnostic group on ICU admission, 1 was gender, 1 was day from ICU admission, and 1 was day of the week.

For the prediction of average CrCl of two days ahead, a total of 149 features were employed in the Core+BGA+Monitoring model. 9 were derived from heart rate, 8 were derived from BGA partial pressure of oxygen, 8 were derived from urea, 8 were derived from BGA pH value, 7 were derived from serum creatinine, 7 were derived from BGA bicarbonate, 7 were derived from BGA sodium, 6 were derived from BGA hemoglobin, 6 were derived from chloride, 6 were derived from BGA potassium, 6 were derived from BGA lactate, 6 were derived from mean arterial blood pressure, 6 were derived from platelet, 6 were derived from SOFA score, 6 were derived from BGA glucose, 5 were derived from BGA partial pressure of carbon dioxide, 4 were derived from respiratory rate, 4 were derived from urine output, 4 were derived from BGA calculated oxygen saturation, 4 were derived from urine creatinine, 4 were derived from hematocrit, 4 were derived from creatinine clearance, 4 were derived from c-reactive protein, 3 were derived from white blood cell count, 2 were derived from medication/intervention, 2 were derived from bilirubin, 1 was age, 1 was weight, 1 was APACHE II score on the first day of ICU, 1 was transplant diagnostic group on ICU admission, 1 was gender, 1 was day from ICU admission, and 1 was day of the week.

24-hour creatininuria

In order to verify the completeness of the urinary collection, we analyzed the weight-based daily creatinine excretion in the whole cohort, and in the male and female patient separately (median and IQR).

- Development cohort:
    - All patients: 13.04 (9.67 – 16.77) mg/kg/24hrs
    - Male patients: 14.60 (10.92 – 18.22) mg/kg/24hrs
    - Female patients: 11.02 (8.27 – 13.75) mg/kg/24hrs
- Validation cohort:
    - All patients: 13.27 (9.55 – 17.43) mg/kg/24hrs
    - Male patients: 14.67 (10.78 – 18.95) mg/kg/24hrs
    - Female patients: 11.33 (8.07 – 14.73) mg/kg/24hrs

Supplementary Figures

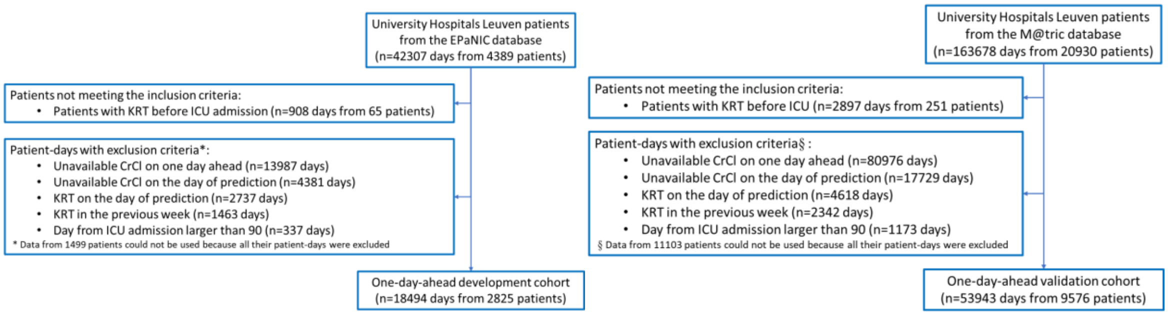

Supplementary Fig. 1 Consort diagram of the development cohort (left) and validation cohort (right) for prediction of one-day-ahead CrCl. CrCl, creatinine clearance; KRT, kidney replacement therapy; ICU, intensive care unit

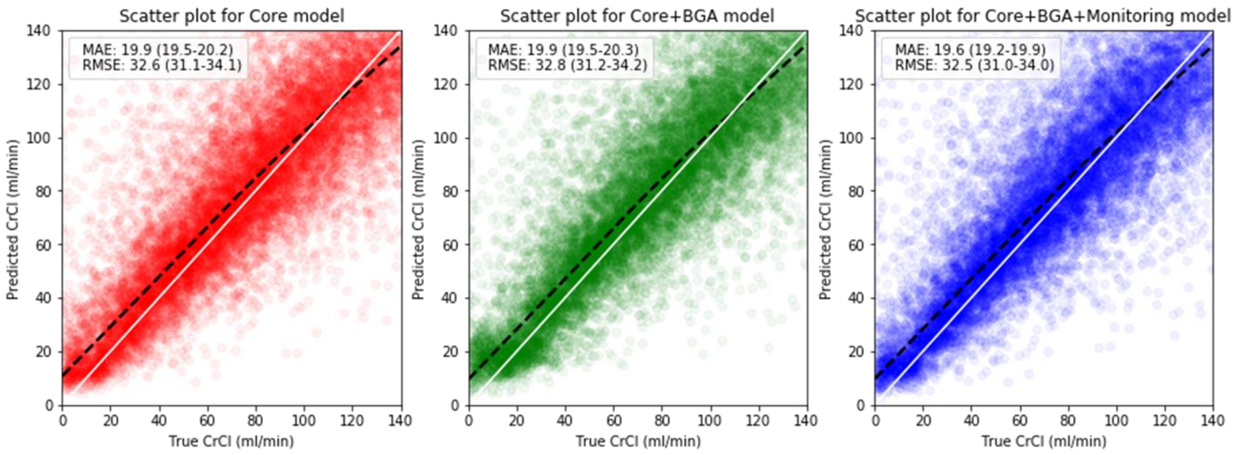

Supplementary Fig. 2 Relationships between predicted and actual CrCls for different models for the one-day-ahead prediction task in the development cohort. The red, green, and blue scatter plots show the results for the Core, Core+BGA, and Core+BGA+Monitoring models. The black dashed and white solid lines represent the lowess-based regression lines for the developed models and the diagonal axis. MAE, mean absolute error; RMSE, root-mean-square error; CrCl, creatinine clearance

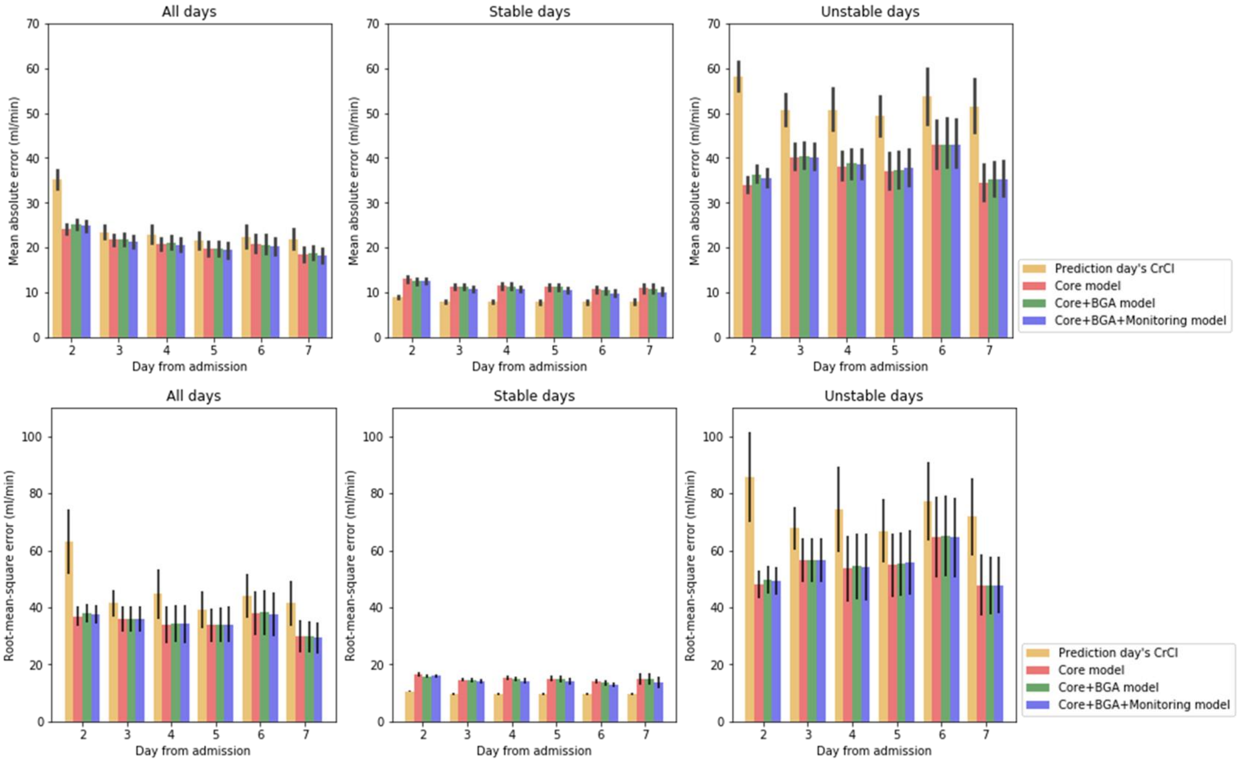

Supplementary Fig. 3 Temporal mean absolute error (upper) and root-mean-square error (lower) of different models on all days, stable days, and unstable days within the first week of ICU admission for the one-day-ahead prediction task in the development cohort. The red, green, blue, and orange bars represent, respectively, the Core, Core+BGA, Core+BGA+Monitoring models, and the reference that assumes CrCl will remain the same compared to the day of prediction. Error bars represent 95% confidence intervals.

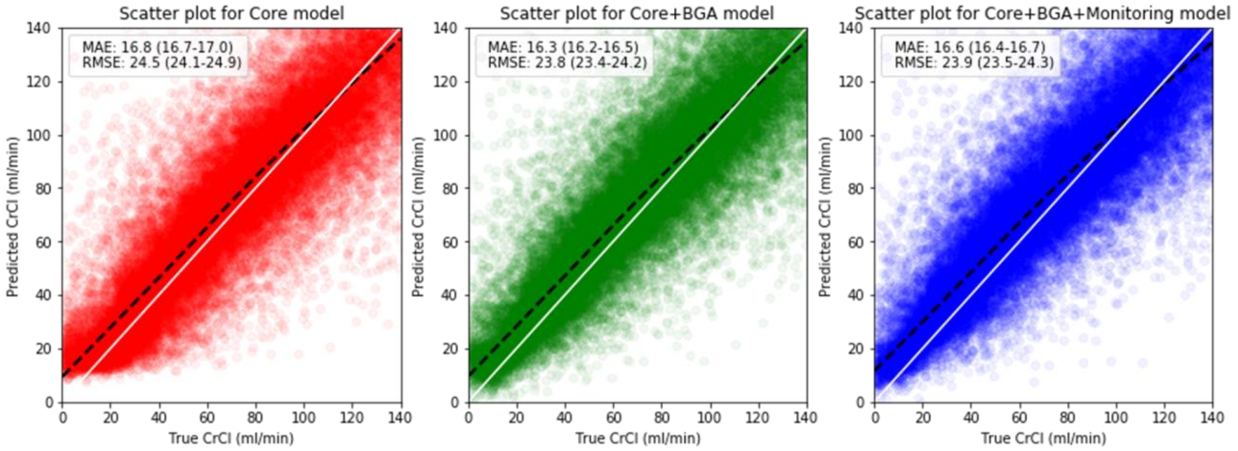

Supplementary Fig. 4 Relationships between predicted and actual CrCls for different models for the average of two days ahead prediction task in the validation cohort. The red, green, and blue scatter plots show the results for the Core, Core+BGA, and Core+BGA+Monitoring models. The black dashed and white solid lines represent the lowess-based regression lines for the developed models and the diagonal axis. MAE, mean absolute error; RMSE, root-mean-square error; CrCl, creatinine clearance

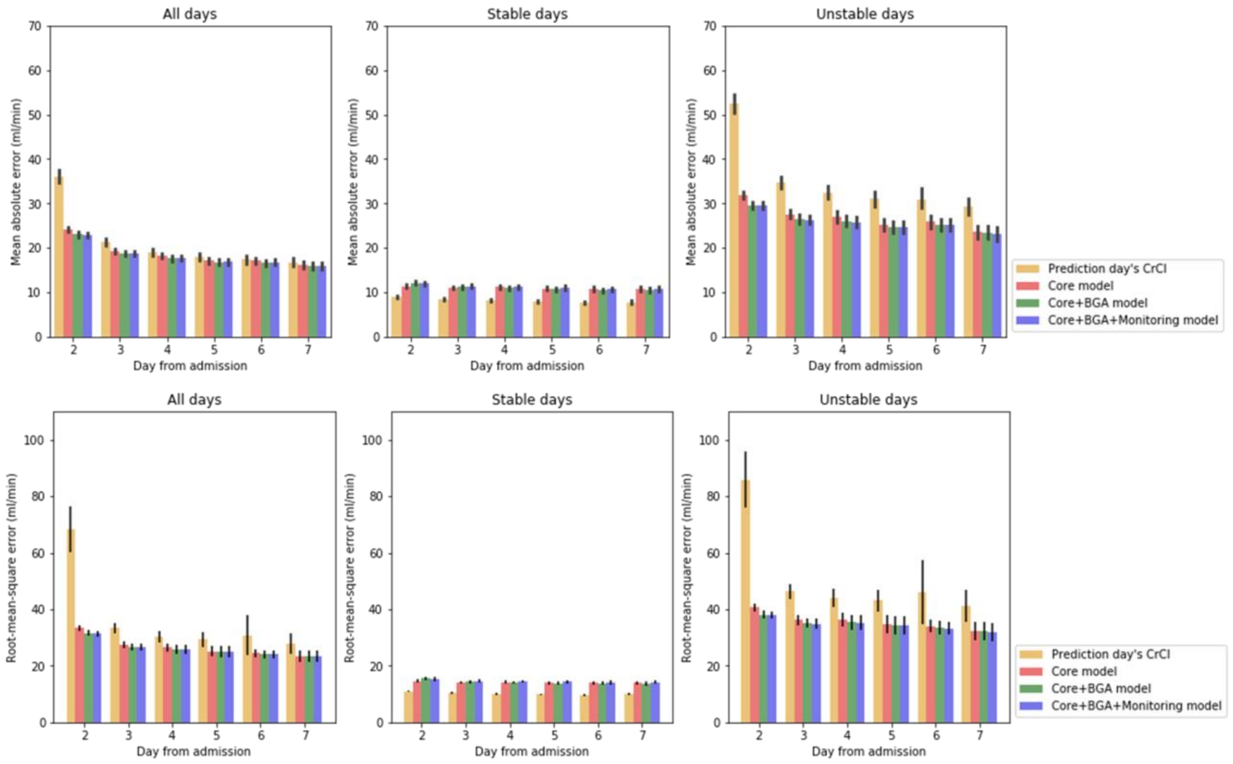

Supplementary Fig. 5 Temporal mean absolute error (upper) and root-mean-square error (lower) of different models on all days, stable days, and unstable days within the first week of ICU admission for the average of two days ahead prediction task in the validation cohort. The red, green, blue, and orange bars represent, respectively, the Core, Core+BGA, Core+BGA+Monitoring models, and the reference that assumes CrCl will remain the same compared to the day of prediction. Error bars represent 95% confidence intervals.

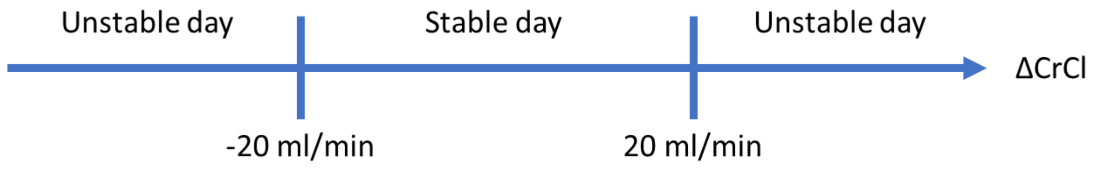

Supplementary Fig. 6 Figure illustrating the stable/unstable days concept. If the absolute ΔCrCl is smaller than 20 ml/min, the study day is labeled as a stable day. If the ΔCrCl is larger than 20 ml/min or lower than -20 ml/min, it is considered unstable. ΔCrCl = (CrCl of the next day-CrCl of the current day. CrCl, creatinine clearance

Supplementary Tables

Supplementary Table 1 Summary of mean absolute error and root-mean-square error for different models and the reference on all days, stable days, and unstable days for the one-day-ahead prediction task in the development cohort

	All days		Stable days		Unstable days	
	Mean absolute error (ml/min) (95% CI)	Root-mean-square error (ml/min) (95% CI)	Mean absolute error (ml/min) (95% CI)	Root-mean-square error (ml/min) (95% CI)	Mean absolute error (ml/min) (95% CI)	Root-mean-square error (ml/min) (95% CI)
**Prediction day's CrCl**	22.5 (22-23)	42.1 (39.3-44.7)	7.8 (7.7-7.9)	9.5 (9.4-9.6)	50.6 (49.4-51.8)	70.6 (65.8-75.1)
**Core model**	19.9 (19.5-20.2)	32.6 (31.1-34.1)	11.1 (10.9-11.3)	14.8 (14.5-15)	36.5 (35.6-37.4)	51.7 (48.9-54.4)
**Core+BGA model**	19.9 (19.5-20.3)	32.8 (31.2-34.2)	10.8 (10.6-11)	14.3 (14-14.5)	37.2 (36.3-38.1)	52.2 (49.4-54.9)
**Core+BGA+Monitoring model**	19.6 (19.2-19.9)	32.5 (31-34)	10.3 (10.2-10.5)	13.7 (13.5-14)	37.1 (36.2-38)	52.1 (49.3-54.8)

CrCl, creatinine clearance
